# Evolution and function of epigenetic processes in the endosperm

**DOI:** 10.3389/fpls.2015.00130

**Published:** 2015-03-09

**Authors:** Claudia Köhler, Clément Lafon-Placette

**Affiliations:** Department of Plant Biology, Uppsala BioCenter, Linnean Center of Plant Biology, Swedish University of Agricultural Sciences, Uppsala, Sweden

**Keywords:** endosperm, evolution, epigenetics, imprinting, Polycomb Repressive Complex 2

## Abstract

The endosperm is an ephemeral tissue surrounding the embryo that is essential for its development. Aside from the embryo nourishing function, the endosperm serves as a battlefield for epigenetic processes that have been hypothesized to reinforce transposable element silencing in the embryo. Specifically, global DNA demethylation in the central cell may serve to produce small RNAs that migrate to egg cell and embryo to induce de novo DNA methylation. The Polycomb Repressive Complex 2 (PRC2) is particularly targeted to DNA hypomethylated regions, possibly alleviating the negative effects associated with loss of DNA methylation in the endosperm. The functional requirement of the PRC2 in the endosperm can be bypassed by increasing the maternal genome dosage in the endosperm, suggesting a main functional role of the endosperm PRC2 in reducing sexual conflict. We therefore propose that the functional requirement of an endosperm PRC2 was coupled to the evolution of a sexual endosperm and mechanisms enforcing transposon silencing in the embryo. The evolutionary consequences of this scenario for genome expansion will be discussed.

## INTRODUCTION

The endosperm is a nutritive tissue formed in seeds of flowering plants that surrounds the embryo and is essential for its development. Embryo and endosperm are the products of two distinct fertilization events and enclosed by the maternally derived integuments that form the seed coat. While the embryo is derived from the fertilized egg cell, the endosperm is the descendent of the fertilized central cell (Li and Berger, 2012). The majority of angiosperms have an eight-nucleated, seven-celled Polygonum-type embryo sack that develops from the single surviving haploid megaspore after three rounds of nuclei divisions, followed by nuclei migrations and cellularization. The female gametophyte consists of the egg cell flanked by two synergid cells, a diploid central cell derived from fusion of two polar nuclei and three antipodal cells that will degenerate shortly before or after fertilization (Sprunck and Gross-Hardt, 2011). As a consequence of the central cell being diploid, the endosperm of most species is triploid. Ancestral to the seven-celled female gametophyte is the four celled female gametophyte that contains a haploid central cell that will form a diploid endosperm after fertilization (Friedman and Williams, 2003; Segal et al., 2003). There are also angiosperms (e.g., Piperaceae) where all four megaspores survive after meiosis and contribute to female gametophyte formation, giving rise to central cells with more than two nuclei that will form a high-ploidy (>3*n*) endosperm after fertilization (Friedman et al., 2008).

## EVOLUTION OF PLOIDY SHIFTS IN THE ENDOSPERM

It has been hypothesized that the transition from a purely maternal embryo nourishing tissue to a biparental endosperm resulted in two possible conflicts: (i) conflict of male and female parents over the allocation of nutrients to the developing progeny and (ii) conflict among the developing progeny for resources from the maternal sporophyte (Haig and Westoby, 1989a,b; Friedman, 1995). The kin-conflict theory provides a theoretical framework for both conflicts (Haig, 2013). This theory considers that the resources provided by the maternal sporophyte to provision the offspring are limited and that the relatedness of the endosperm to the maternal sporophyte is decisive for its ability to acquire nutrients for the developing embryo. In outcrossing species, the sexual endosperm containing maternal and paternal genomes is less related to the maternal sporophyte and sibling embryos compared to its own embryo (Charnov, 1979; Friedman et al., 2008) Therefore, paternally contributed alleles maximizing nutrient allocation to the endosperm will promote development of the embryo supported by this genetically related endosperm on the expense of the sibling embryos (Haig and Westoby, 1989a,b; Haig, 2013). Increasing the number of maternal genomes contributed to the central cell could have evolved as a mechanism to control resource provisioning to the developing progeny and to limit the selfish behavior of the endosperm. Therefore, transitions from the haploid central cell to the diploid and higher ploidy central cell can be viewed as evolutionary transitions to resolve conflict between maternal and paternal genomes on the provisioning of the progeny (Friedman et al., 2008).

## EPIGENETIC PROCESSES IN THE ENDOSPERM

In *Arabidopsis*, DNA methylation occurs in CG, CHG, and CHH (where H = A, T, or C) sequence contexts and is controlled by three families of DNA methyltransferases that have different sequence preference. The DNA methyltransferase MET1 acts as a maintenance methyltransferase for symmetric CG residues, while non-CG methylation is maintained by the CHROMOMETHYLASE3 (in CHG context) and the DOMAINS REARRANGED METHYLASES 1/2 (DRM1/2) and CMT2 (in CHH context). The small RNA (sRNA) pathway targets DRM1/2-mediated *de novo* methylation in all sequence contexts and is required for the maintenance of CHH methylation (Kim and Zilberman, 2014). Genome-wide methylation studies of embryo and endosperm in *Arabidopsis* seeds revealed that the endosperm is globally hypomethylated compared to the embryo (Gehring et al., 2009; Hsieh et al., 2009), signifying substantial epigenome differences of the two fertilization products. Hypomethylation in the endosperm is restricted to the maternally inherited alleles, suggesting that the hypomethylated status is established in the central cell and inherited to the endosperm (Ibarra et al., 2012). In *Arabidopsis*, demethylation of the maternal genome requires the DNA glycosylase DEMETER (DME) that excises 5-methylcytosine preferably at small transposable elements (TEs) and is expressed in the central cell of the female gametophyte before fertilization (Hsieh et al., 2009). Maternal demethylation is nearly fully reversed in *dme* mutant endosperm (Ibarra et al., 2012), indicating that DME is likely the only enzyme accounting for global DNA methylation differences between the maternal and paternal endosperm genomes in *Arabidopsis*. The process of extensive endosperm demethylation is likely conserved between monocots and dicots, but involves specific differences caused by divergent evolution of the DME-like family (Zemach et al., 2010). Importantly, DME function is not restricted to the female central cell but DME also acts in the vegetative cell in pollen, the companion cell to the sperm cells. Similar to its role in the central cell, DME is causing hypomethylation of distinct regions in the vegetative cell. Almost half of those hypomethylated regions in the vegetative cell overlap with hypomethylated regions identified in the maternal genomes of the endosperm that likely descend from the central cell (Ibarra et al., 2012). While *de novo* methylation in CHH context is generally depleted in sperm (Calarco et al., 2012; Ibarra et al., 2012), regions that become hypomethylated by DME in the vegetative cell have increased levels of CHH methylation in sperm (Ibarra et al., 2012), suggesting communication between vegetative cells and sperm cells. Thus, it seems likely that sRNAs that are formed from demethylated regions in the vegetative cell migrate to sperm cells and reinforce methylation at distinct target sites, a hypothesis that remains to be experimentally tested. In agreement with this notion, 21 nt and 24 nt sRNAs corresponding to differentially methylated regions accumulate in sperm cells (Slotkin et al., 2009; Calarco et al., 2012) and prominently target regions of imprinted genes (discussed below) that maintain the paternal allele silenced after fertilization (Calarco et al., 2012). While traveling of a micro-RNA from the vegetative cell to sperm cells was proposed (Slotkin et al., 2009), this study may have suffered from an unspecific promoter (Grant-Downton et al., 2013), biasing the conclusions. Nevertheless, expression of a micro-RNA in the central cell can silence a reporter in the egg cell (Ibarra et al., 2012); revealing that 21 nt sRNAs (that are preferentially formed from microRNAs) can indeed travel from the companion cells to the neighboring gametes. While previously only 24 nt sRNAs were known to establish *de novo* methylation by the sRNA-dependent DNA methylation pathway (RdDM; Cao and Jacobsen, 2002; Cao et al., 2003), recent data revealed a role of 21 nt sRNAs in silencing of transcriptionally active TEs via the RdDM pathway (McCue et al., 2015).

*De novo* DNA methylation increases during embryo development, suggesting increased activity of the RdDM pathway during embryo development (Jullien et al., 2012). In agreement with this view, *de novo* DNA methylation in the embryo depends on the activity of DRM2 and in part as well on DRM1 (Jullien et al., 2012). After fertilization, increased production of sRNAs occurs in siliques, reaching maximum levels at 6 days after anthesis (Mosher et al., 2009). Increased sRNA production correlates with a steadily increasing *de novo* methylation in the embryo (Jullien et al., 2012), giving rise to the hypothesis that sRNAs migrate from the endosperm to the developing embryo and enforce TE silencing in the embryo by de novo DNA methylation. *De novo* DNA methyltransferases DRM1 and DRM2 seem not to be active in the early endosperm (Jullien et al., 2012) but are expressed around the time of endosperm cellularization (Belmonte et al., 2013), in agreement with the presence of substantial levels of CHH methylation levels in the cellularized endosperm (Hsieh et al., 2009; Ibarra et al., 2012).

## GENOMIC IMPRINTING IN THE ENDOSPERM

As a consequence of DNA hypomethylation in the central cell, the parental genomes are differentially methylated in the endosperm, which can cause genes to become preferentially expressed from either the maternally or paternally inherited alleles. Parent-of-origin dependent gene expression as a consequence of epigenetic modification of maternal and paternal alleles in the gametes is a well-known phenomenon termed genomic imprinting (Gehring, 2013). Hypomethylation of TEs can cause either activation or silencing of the neighboring genes. What determines whether a gene will become activated or silenced in response to hypomethylation remains to be resolved, however, it seems likely that the distance of the TE to the gene is decisive. While many maternally and paternally expressed imprinted genes (MEGs and PEGs, respectively) have TEs in the vicinity of the 5′ region, many PEGs have TEs additionally in the coding and 3′ region of the gene (Wolff et al., 2011; Ibarra et al., 2012). Hypomethylation might expose binding sites for the repressive FERTILIZATION INDEPENDENT SEED (FIS)-Polycomb Repressive Complex 2 (PRC2), as it has been proposed for the differentially methylated region downstream of the *PHERES1* gene (Villar et al., 2009). The PRC2 is an evolutionary conserved repressive complex that modifies histones by applying histone trimethylation marks on histone H3 at lysine 27 (H3K27me3; Simon and Kingston, 2013). In *Arabidopsis*, there are at least three PRC2 complexes with different functional roles during plant development. The FIS-PRC2 is specifically expressed in the central cell and in the endosperm and consists of the subunits MEDEA (MEA), FIS2, FERTILIZATION INDEPENDENT ENDOSPERM (FIE), and MSI1 (Hennig and Derkacheva, 2009). Genome-wide profiling of H3K27me3 occupancy in the endosperm revealed that several DNA hypomethylated TEs were targeted by the FIS-PRC2, revealing a redistribution of the FIS-PRC2 dependent on the location of DNA methylation (Weinhofer et al., 2010). A similar redistribution of H3K27me3 to TEs occurs in mutants deficient for MET1 (Deleris et al., 2012), as well as in mammalian cells depleted for DNA methylation (Reddington et al., 2013; Saksouk et al., 2014) revealing a general ability of PRC2 to target and possibly silence hypomethylated TEs.

## EVOLUTION OF EPIGENETIC PROCESSES IN THE ENDOSPERM AS A MECHANISM TO PREVENT GENOME EXPANSION

Thus far, we do not know when endosperm-expressed PRC2 genes have emerged during angiosperm evolution. While the FIS-PRC2 subunit encoding genes *FIS2* and *MEA* are specific for *Arabidopsis thaliana* (Spillane et al., 2007; Chen et al., 2009), functional homologs of both genes are expressed in the triploid endosperm of monocots and lower eudicots (Haun et al., 2007; Luo et al., 2009; Gleason and Kramer, 2012) suggesting that the evolution of FIS-PRC2-like complexes is connected with the evolution of a sexual endosperm. Nevertheless, homologs but not orthologs fulfill the FIS2-PRC2 functional role in dicots and monocots and different PRC2 genes are regulated by genomic imprinting in monocots (Danilevskaya et al., 2003; Luo et al., 2009) and dicots (Grossniklaus et al., 1998; Luo et al., 2000), raising the hypothesis that FIS-PRC2-like complexes have evolved independently in both plant groups. The functional requirement of the FIS-PRC2 can be bypassed by increasing the maternal genome dosage in the endosperm (Kradolfer et al., 2013), suggesting that the FIS-PRC2 serves to suppress expression of paternally contributed genes. Supporting this view, *Arabidopsis* mutants lacking a FIS-PRC2 can form a functional diploid endosperm (Nowack et al., 2007); revealing that by reducing paternal genome dosage the functional requirement of the FIS-PRC2 can be bypassed. We therefore hypothesize that in gymnosperms, where the female gametophyte forms an endosperm-like nourishing structure, a FIS-PRC2-like complex would not be required. This notion is supported by the fact that the FIS-PRC2 prevents autonomous endosperm formation and thus couples fertilization to endosperm development (Guitton and Berger, 2005), a function that is not required in gymnosperms.

**FIGURE 1:**
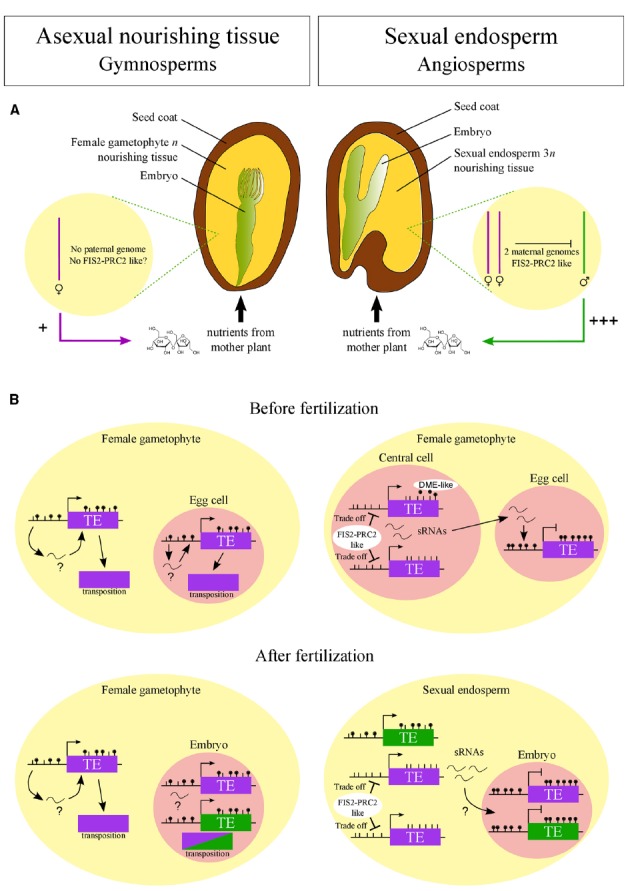
**Hypothesized emergence of FIS2-PRC2-like complex in angiosperms connected to its role in sexual endosperm. (A)** In angiosperms, FIS2-PRC2-like function may have arisen to repress the paternal genome in the endosperm as predicted by the kin-conflict theory. The selfish behavior of the paternal genome, promoting nutrient allocation to the progeny, is symbolized by “+++”. According to this hypothesis, in gymnosperms, where the nourishing tissue is purely maternal, FIS2-PRC2-like function is not required. **(B)** FIS2-PRC2-like function may have allowed the emergence of DNA hypomethylation via DME-like activity and small RNA (sRNA) production in central cell and endosperm. FIS2-PRC2 acts on hypomethylated regions, limiting the deleterious activity of transposable elements (TE). This tradeoff between TE silencing and activity allows the production of sRNAs traveling to the egg cell and embryo, reinforcing TE silencing. Such a demethylation process may not have emerged in gymnosperms, leading to a limited silencing of TEs and explaining the genome expansion in this taxon. Purple and green colors symbolize maternal and paternal genomes, respectively.

As outlined above, the PRC2 is targeted to regions with reduced DNA methylation in the endosperm (Weinhofer et al., 2010; Zhang et al., 2014) and could, therefore, potentially repress activity of TEs upon loss of DNA methylation. It can thus be envisioned that DNA hypomethylation activities in the central cell evolved concomitantly with a central cell/endosperm-expressed PRC2 that alleviated the negative effects associated with loss of DNA methylation. Following this logic, the evolution of hypomethylation mechanisms in the central cell to enforce silencing of TEs in the egg cell and descendent embryo were closely coupled to the evolution of FIS-PRC2-like complexes in the central cell and endosperm to ensure TE silencing after DNA hypomethylation. This scenario would imply that TE transcription upon DNA hypomethylation and PRC2-mediated TE repression are balanced to ensure sufficient TE-derived sRNAs being made to enforce silencing in the embryo but TE transposition remains suppressed (Figure 1).

If indeed a FIS-PRC2-like complex as well as DME-like enzymes evolved together with the sexual endosperm, neither of both should be present in the large female gametophytes of gymnosperms. The female gametophyte in gymnosperms serves an endosperm-like role in supporting embryo growth, but it is of pure maternal origin and forms a multicellular structure before fertilization. Consequently, the enormous genome expansion in gymnosperms may be a consequence of the lack of efficient TE silencing mechanisms that possibly have evolved after the female gametophyte became sexualized (Figure 1).

Testing this hypothesis requires to test whether orthologs of FIS-PRC2-like genes and DME-like genes are expressed in the female gametophyte of gymnosperms and basal angiosperms, which remains a challenge of future investigations.

Together, we propose that the evolution of mechanisms enforcing TE silencing in the embryo evolved concomitantly with the sexual endosperm. The evolution of PRC2 activity in the central cell and endosperm allowed DNA hypomethylation activities being active in the central cell to enforced TE silencing in the egg cell and embryo. Consequently, genome size restriction by efficient control of TE silencing may be directly coupled with the evolution of a sexual endosperm.

### Conflict of Interest Statement

The authors declare that the research was conducted in the absence of any commercial or financial relationships that could be construed as a potential conflict of interest.
